# Response Factorial Design Analysis on Papain-Generated Hydrolysates from *Actinopyga lecanora* for Determination of Antioxidant and Antityrosinase Activities

**DOI:** 10.3390/molecules25112663

**Published:** 2020-06-08

**Authors:** Aqilah Noor Bahari, Nazamid Saari, Norazlinaliza Salim, Siti Efliza Ashari

**Affiliations:** 1 Halal Products Research Institute, Putra Infoport, Universiti Putra Malaysia, Serdang 43400 UPM, Selangor, Malaysia; aqilahnoorbahari@gmail.com; 2 Integrated Chemical BioPhysics Research, Faculty of Science, Universiti Putra Malaysia, Serdang 43400 UPM, Selangor, Malaysia; ctefliza@upm.edu.my; 3 Department of Food Science, Faculty of Food Science and Technology, Universiti Putra Malaysia, Serdang 43400, Selangor, Malaysia; 4 Centre of Foundation Studies for Agricultural Science, Universiti Putra Malaysia, Serdang 43400 UPM, Selangor, Malaysia

**Keywords:** factorial design optimization, hydrolysates, antioxidant, antityrosinase, *Actinopyga lecanora*

## Abstract

*Actinopyga lecanora* (*A. lecanora*) is classified among the edible species of sea cucumber, known to be rich in protein. Its hydrolysates were reported to contain relatively high antioxidant activity. Antioxidants are one of the essential properties in cosmeceutical products especially to alleviate skin aging. In the present study, pH, reaction temperature, reaction time and enzyme/substrate ratio (E/S) have been identified as the parameters in the papain enzymatic hydrolysis of *A. lecanora*. The degree of hydrolysis (DH) with antioxidant activities of 2,2-diphenyl-1-picrylhydrazyl (DPPH) and ferric-reducing antioxidant power (FRAP) assays were used as the responses in the optimization. Analysis of variance (ANOVA), normal plot of residuals and 3D contour plots were evaluated to study the effects and interactions between parameters. The best conditions selected from the optimization were at pH 5.00, 70 °C of reaction temperature, 9 h of hydrolysis time and 1.00% enzyme/substrate (E/S) ratio, with the hydrolysates having 51.90% of DH, 42.70% of DPPH activity and 109.90 Fe^2+^μg/mL of FRAP activity. The *A. lecanora* hydrolysates (ALH) showed a high amount of hydrophobic amino acids (286.40 mg/g sample) that might be responsible for antioxidant and antityrosinase activities. Scanning electron microscopy (SEM) image of ALH shows smooth structures with pores. Antityrosinase activity of ALH exhibited inhibition of 31.50% for L-tyrosine substrate and 25.40% for L-DOPA substrate. This condition suggests that the optimized ALH acquired has the potential to be used as a bioactive ingredient for cosmeceutical applications.

## 1. Introduction

Aging is one of the natural processes of human skin upon time. It is described by an accretion of molecular damage and a gradual failure of maintenance and repair. Wrinkles, loss of elasticity, thinning and freckles are the visible signs of skin aging. Women aged 40 and above tend to age predominantly as the thickness of the skin and collagen is decreased naturally. Moreover, with a chronic contact of UV radiation on skin, results in solar elastosis and degradation of the extracellular matrix (ECM) [1].

Antioxidants are reported to have the tendency to prevent cells from oxidative stress by scavenging the reactive oxygen species (ROS) or free radicals. The antioxidant will neutralize the free radicals by combining with oxygen molecules in the destabilization process [2]. Apart from that, bioactive peptides or biopeptides are one of the latest elements in skin care formulations. To slow down the aging processes, antiaging cosmetic biopeptides have been produced and have attracted considerable attention in the cosmetic industry [3].

Another good trait present in cosmeceutical products is the antityrosinase activity. Tyrosinase is an oxidase containing copper that is widely distributed in microorganisms, plants and animals [4]. Consequently, tyrosinase is the key enzyme in melanin biosynthesis that is typically correlated with skin problems such as hyperpigmentation, age spots and freckles. The build-up of the melanin pigment in hyperpigmentation can be treated with depigmenting agent or tyrosinase inhibitors [5]. Therefore, the quest for potent tyrosinase inhibitors has led to numerous screened tests from plants and animal natural sources for example proteins and peptides [6,7]. Nakchum and Kim [4] found out that an F3 fragment from squid skin collagen hydrolysates inhibited tyrosinase activity by 39.70% at 1 mg/mL, and the activity was reported to be related with the Cu^2+^ chelating ability of the hydrolysates. Besides that, Karkouch et al. [6] reported on the synthesized peptide fragments from plant *Vicia faba* seed protein hydrolysates for their ability to inhibit tyrosinase activity by using L-tyrosine and L-DOPA as substrates with the inhibition recorded at 60.00% and 22.00%, respectively.

Likewise, biopeptides are smaller peptides with 2–20 amino acids present in the protein hydrolysates, which is generated through hydrolysis. They are biologically active and can stimulate natural biological processes that can help reverse the signs of aging. They also supply the second stage of necessary biological materials in the form of macromolecules, protein precursors and peptides for the formation of larger complex proteins, prostaglandins and hormones. This means that biopeptides are capable of stimulating the physiological processes within the skin itself [1].

Hydrolysates are the complex mixture of oligopeptides, peptides and free amino acids produced from numerous protein sources (plant and animal) via several processes such as chemical hydrolysis, microbial fermentation and enzymatic hydrolysis [8]. Among the methods, enzymatic hydrolysis is the most widely used process for hydrolysates production. This process beholds several advantages, such as it requires only milder condition process (pH 5.00–8.00; temperature 40–70 °C) with short reaction times (4–9 h), better predictability control for hydrolysis and does not involve organic solvent or toxic chemicals [9]. The process also can be reproduced based on its specific protease used [10].

The source of hydrolysates used in this research comes from *Actinopyga lecanora*, an edible marine invertebrate species of sea cucumber. It is commonly found in Malaysia and other South Asian countries as by-catch in the fishery industry. Despite being abundantly available, cheap sources, easy to propagate with great commercial potential, this species is still underutilized, especially in cosmeceutical applications. Previous researchers have recently reported the generation of antioxidant peptides from *A. lecanora* hydrolysates that can be categorized among the potential sources of functional foods [11]. The antioxidant activity was discovered to be dependent on the enzyme specificity, degree of hydrolysis and peptide sequences generated [12]. Thus, the selection of the optimum condition of hydrolysis is crucial to obtain maximum antioxidant activity.

In another work, Ghanbari et al. [13] reported on the antibacterial activity of *A. lecanora* hydrolysates against Gram-positive bacteria and Gram-negative bacteria. Bromelain hydrolysates exhibited the highest antibacterial activities against Gram-negative bacteria at 52.00%. Moreover, antihypertensive activities of alcalase hydrolysates from *A. lecanora* were successfully reported to have 69.80% of 2,2-diphenyl-1-picrylhydrazyl (DPPH) radical scavenging activity [14].

Based on previous works related to the *A. lecanora* hydrolysates, no studies have been reported on the optimization of papain enzymatic hydrolysis towards antioxidants and antityrosinase activity. Thus, in the present study, a two-level full-factorial design was carried out to determine the best overall condition for papain hydrolysis of *A. lecanora*. The benefit of this approach is it can achieve the most information in the least number of runs, as all factor-level combinations can be detected [15]. The four factors responsible for enzymatic hydrolysis were the pH, reaction time, reaction temperature and enzyme/substrate (E/S) ratio, while the responses determined were the degree of hydrolysis (DH), 2,2-diphenyl-1-picrylhydrazyl (DPPH) activity and ferric-reducing antioxidant power (FRAP) activity.

## 2. Materials and Methods

### 2.1. Materials

Fresh *A. lecanora* samples were obtained from Kedah and Langkawi Breeding Centers (Malaysia). Papain enzyme was purchased from Acros Organics (St. Louis, MO, USA). Potassium dihydrogen phosphate, dipotassium hydrogen phosphate, sodium acetate, iron (II) chloride tetrahydrate, phenyl isothiocyanate and glacial acetic acid were obtained from Merck (Darmstadt, Germany). *O*-phthaldialdehyde (OPA), dithiothreitol (97%) and α-aminobutyric acid were purchased from Sigma–Aldrich (Buchs, Switzerland), 2,2-diphenyl-1-picrylhydrazyl (DPPH) was purchased from Sigma-Aldrich (St. Louis, MO, USA) and 2,4,6-tris(2-pyridyl)-s-triazine (TPTZ) was purchased from Fluka (Buchs, Switzerland). Deionized water was from a purified Milli-Q water system (EMD Millipore) from Billerica, MA, USA.

### 2.2. Preparation of Hydrolysates from A. Lecanora

Preparation of *A. lecanora* hydrolysates was conducted according to Auwal et al. [16], with minor modifications. One kilogram of *A. lecanora* sample was freeze-dried, ground using a Waring^®^ blender (Stamford, CT, USA) and sieved with a 600-μm strainer. Five grams of powdered sample was dialyzed in a 12–14 kDa molecular mass cut-off dialysis tube for 24 h at 4 °C (4 h in deionized water and 20 h in a buffer solution, pH 5.00 or 8.00). After dialysis, the sample was mixed with 150 mL of 50-mM buffer solution and subjected to hydrolysis under different reaction conditions (pH, reaction temperature, reaction time and enzyme/substrate ratio), according to Table 1. The sample mixture was then preheated to the desired temperature, and papain enzyme was added (0.50–5.00% E/S ratio). The hydrolysis was carried out at 150 rpm in a shaker bath (PROTECH, Model 903). To terminate the hydrolysis reaction, the mixture was heated in 100 °C boiling water for 10 min. The mixture was then centrifuged for 20 min at 4 °C at 10,000 rpm. The supernatant was collected, frozen overnight in a −80 °C freezer before subjected to a freeze drier at −109 °C and continuously used for further study.

### 2.3. Experimental Design

In the optimization of the enzymatic hydrolysis of *A. lecanora*, a 2^k^ full-factorial experimental design was developed by Design Expert 7.0 software^®^ (Version 7.1.6, Stat Ease Inc., Minneapolis, MN, USA). The k factors were examined at two levels, high and low or +1 and −1, respectively. The design was implemented to reduce the total number of experiments to achieve optimized conditions [17]. In the present study, four parameters were identified and used as independent variables: pH, reaction temperature (°C), reaction time (t) and enzyme/substrate (E/S) ratio (%) toward the responses, degree of hydrolysis (%), DPPH activity (%) and FRAP activity (Fe^2+^μg/mL). The ranges of two levels were set based on screening experiments and a literature study [11,16]. The statistical results from the experiment of the model were analyzed by analysis of variance (ANOVA) with a *p* value < 0.05 considered significant. A summary of the independent variables with coded levels is tabulated in Table 1.

### 2.4. Statistical Analysis and Model Verification

In the experimental design, to demonstrate the relationship between factors and responses, a regression model was used in terms of a mathematical equation for each response. The mathematical equation was also essential in understanding the level and effect of each parameter [18]. The general regression equation was expressed as Equation (1):

(1) $$Y=z_{0}+\sum z_{1}A+\sum z_{12}AB+\sum z_{123}ABC+\sum z_{1234}ABCD$$

where z_0_ was the mean value of the model; z_1_ was the coefficient of parameter A and z_12_, z_123_ and z_1234_ were the coefficients of the interaction parameters for AB, ABC and ABCD, respectively.

Analysis of variance (ANOVA) and the R^2^ coefficient were used to investigate the significant differences among the independent variables. In ANOVA, the *p*-value of the model must be significant with *p* < 0.05 and an R^2^ higher than 0.9 in order to get a good final reduced model. A few sets of random independent variables were constructed to validate the model.

The actual value was compared with the predicted value by calculating the residual standard error (RSE), as in Equation (2). The RSE value less than 5.00% indicated no significant difference between the actual and predicted value [19,20]:

(2) $$Residual\;standard\;error\left(\%\right)=\left|\frac{Actual\;value-Predicted\;Value}{Predicted\;value}\right|\times 100$$

### 2.5. Response Parameter Analysis

#### 2.5.1. Determination of Degree of Hydrolysis

The degree of hydrolysis (DH) was piloted based on the *o*-phthaldialdehyde (OPA) method used by Auwal et al. [12] and Nielsen et al. [21], with slight modifications. The OPA reagent was freshly prepared by dissolving 7.620 g of sodium tetraborate decahydrate (Na_2_[B_4_O_5_(OH)_4_]·8H_2_O) and 0.200 g of sodium dodecyl sulfate (NaC_12_H_25_SO_4_) in 150 mL deionized water (Solution 1). Then, 0.160 g of *o*-phthaldialdehyde (C_8_H_6_O_2_) was dissolved in 4 mL of ethanol (Solution 2), while 0.176 g of dithiothreitol (C_4_H_10_O_2_S_2_) was dissolved in 5 mL of deionized water (Solution 3). All solutions (1, 2 and 3) were mixed in a volumetric flask and filled up with deionized water up to 200 mL. A 150 μL OPA reagent was added to 50 μL samples in a 96-well microplate, incubated for exactly 2 min and the absorbance was read at 340 nm. The alpha-amino group content of the sample was determined as the concentration of L-serine from a standard curve, prepared as follows: 50 mg L-serine was dissolved in 500 mL deionized water. The DH was then calculated using following Equation (3):

(3) $$DH\left(\%\right)=\frac{L_{t}-L_{0}}{L_{total}-L_{0}}\times 100$$

where L_t_ was the amount of free amino groups discharged in hydrolysis at a certain time t, L_0_ was the amount of free amino groups at t = 0 and L_total_ was the total amount of free amino groups in the original sample acquired from complete acid hydrolysis with 6.0 M HCl for 24 h at 110 °C.

#### 2.5.2. Determination of Antioxidant Activities

DPPH Radical Scavenging Activity: The DPPH radical scavenging activity was determined according to the method explained by Hwang et al. [22], with minor modifications. One-hundred microliters of freshly prepared 0.25 mM DPPH in methanol was added to a 100 μL sample in a 96-well microplate. The mixture was then incubated for 30 min in the dark, and the absorbance was determined at wavelength 517 nm using an ELISA microplate reader (Labomed, model UVD-2950, USA). The scavenging activity was calculated based on the following Equation (4):

(4) $$DPPH\left(\%\right)=\left[1-\frac{\left(A_{s}-A_{b}\right)}{\left(A_{c}\right)}\right]\times 100$$

where A_s_ was the absorbance of the sample, A_b_ was the absorbance of blank and A_c_ was the absorbance of control.

Ferric-Reducing Antioxidant Power Activity: The FRAP activity was conducted based on the method proposed by Ismail et al. [23], with minor modifications. The working FRAP reagent was freshly prepared by mixing 100 mL of 300-mM acetate buffer pH 3.6, 10 mL of 40-mM HCl containing 10-mM 2,4,6-tripyridyl-s-triazine (TPTZ) solution and 10 mL of 20-mM FeCl_3_·6H_2_O solution. A 3 mL aliquot of FRAP was added to a 100-μL sample and incubated in 37 °C for 30 min. The absorbance was read at wavelength 593 nm against a blank. The FRAP activity was expressed as the concentration of antioxidants having a ferric-reducing ability equivalent to the 1-mg/mL FeSO_4_·2H_2_O standard.

### 2.6. Amino Acid Composition of the Optimized A. lecanora Hydrolysates

Amino acid composition was studied by adapting the procedure used by Wan Mohtar et al. [24], with some modifications. The compositions were analyzed by the Waters HPLC (Hitachi Instrument, Japan) system with a photodiode array detector (MD-2010, Japan). A 0.089 g of optimized *A. lecanora* hydrolysates was added to 15 mL 6 N HCl and purged with nitrogen gas for 1 min. The mixture was fully hydrolyzed in an oven at 100 °C for 24 h. After that, 10 mL of α-aminobutyric acid (AABA) was added as the internal standard. The mixture was transferred to a 50 mL volumetric flask and made up with deionized water. The sample was then derivatized with phenyl isothiocyanate (PITC).

The derivatized sample was dissolved in 100-μL buffer A (0.1-M ammonium acetate, pH 6.5). A 20 μL of prepared sample was injected into an HPLC system using a gradient system of buffer A (0–100% after 5 min) and buffer B (0–100% after 50 min). Buffer B consists of 0.1-M ammonium acetate containing acetonitrile and methanol with a 44:46:10 v/v ratio, pH 6.50. The reverse-phase column used was from Thermal (C18, 5 μ, 250 x 4.6 mm), and the operating temperature was 43 °C. The absorbance at 254 nm was applied for the calculations. The identification of amino acids was determined by comparing the retention times of the standard amino acid mixture (Sigma). The results were analyzed by the Borwin chromatography software (Version 1.5, Jasco Co. Ltd., Japan).

### 2.7. Scanning Electron Microscopy

Scanning electron microscopy (SEM) was applied to investigate the morphology of the optimized hydrolysates. SEM was carried out using a Philips XL-30 instrument (Philips, Eindhoven, The Netherlands). The sample was mounted thinly on a strip of self-adhesive carbon paper. The sample was then sputter-coated with gold and observed at an acceleration voltage of 10 kV.

### 2.8. Determination of Tyrosinase Inhibition Activity

Tyrosinase inhibition activity was carried out according to Nakchum et al. [4], with some modifications. A 9.31 mg of tyrosinase enzyme was dissolved in 25 mL of 50-mM phosphate buffer solution to achieve a concentration at 1000 U/mL. A L-DOPA substrate was prepared by dissolving 0.039 g of L-3.4-dihydroxyphenylalanine in 100 mL of 50-mM phosphate buffer, while a L-tyrosine substrate was prepared by dissolving 0.036 g of L-tyrosine in 100 mL of 50-mM phosphate buffer. A 50 μL sample was pipetted into a 96-well microplate reader. A 264 μL of substrate was added and incubated for 10 min. Then, 6 μL of the tyrosinase enzyme was added to the mixture and incubated for another 20 min. The absorbance was read at 492 nm. Kojic acid was used as the positive control. The tyrosinase inhibitory activity was calculated in Equation (5):

(5) $$Percent\;inhibitory=\frac{\left(C-D\right)-\left(A-B\right)}{\left(C-D\right)}\times 100$$

where A and C were the absorbances of the sample and control, while B and D were the absorbances of the control blank and sample blank, respectively. Each experiment was carried out in triplicate.

## 3. Results and Discussion

### 3.1. Model Fitting and Analysis of Variance

A two-level factorial design was applied to investigate the effects of pH, reaction temperature, reaction time and enzyme/substrate (E/S) ratio on the DH, DPPH radical scavenging and FRAP activity on *A. lecanora* hydrolysates. Table 2 shows the design matrix with the actual and predicted values of each response. All analyses were conducted in triplicate.

The actual values ranged from 19.10–91.70%, 23.30–47.30% and 70.60–141.60 Fe^2+^μg/mL for DH, DPPH radical scavenging activity and FRAP activity, respectively. The effects of these independent variables towards their responses were investigated statistically using analysis of variance (ANOVA).

Table 3 displays the analyzed results of ANOVA for all three different responses. The coefficient of determination (R^2^) for DH, DPPH radical scavenging activity and FRAP activity were 0.9948, 0.9979 and 0.9968, respectively. Moreover, the predicted R^2^ (pred R^2^) value for all responses also represent the high, fitted with 0.9162, 0.9667 and 0.9491 for DH, DPPH radical scavenging and FRAP activity, respectively. The pred R^2^ value showed how well a regression model can predict their response values [25]. According to Gottipati et al. [17] and Bordbar et al. [26], the R^2^ value above 0.9 designated that there is a good fit with high correlation between the experimental values and the regression model. This indicated that the model could describe more than 99% of the response’s variables. 

Apart from that, the significance model also can be described in the F values and *p*-value. The F values for DH, DPPH radical scavenging and FRAP activity were 69.08, 174.48 and 113.87, respectively, while the *p*-value was relatively low in all responses, with less than 0.05, thus indicating the significance model. As noted by Elhalil et al. [15], a large F value with low *p*-value signifies that the independent variables have a significant impact on their respective responses. 

Based on the lowest *p*-value (<0.0001) of independent variables in Table 3, pH (A) gave the most significant impact on DH and DPPH radical scavenging responses. This was followed by the E/S ratio (D), which had an impact on DPPH radical scavenging and FRAP activity. The reaction time (C) was also significant towards the DPPH radical scavenging response. These significant variables were important in constructing a model that agrees with all the responses. The constructed model shows good agreement between the predicted and corresponding experimental results [27].

Data from the ANOVA was used to acquire the best-fitting mathematical model, as given in Table 4. For example, the value of coefficient ABD in the Y_1_ response was found to be insignificant; hence, it was removed from the regressed equation. Other significant coefficients were kept in the equation. So, after discarding the insignificant coefficients, the final reduced model had only the significant main and interaction effects of the variables, selected based on their probability (*p*-value) with *p* < 0.05 [15].

The negative symbol before each coefficient indicated that the parameter gave a negative effect to the response, while the positive symbol gave positive effect [17]. In Equations (6) and (7), the magnitude of the coefficient for A was negative, while the magnitude of coefficients for C and D were positive. This suggested that DH and DPPH radical scavenging activity were inversely proportional to the pH (A) and directly proportional to the reaction time (C) and E/S ratio (D). B coefficient was insignificant, so that it was removed from the equation. Similar to the response equation for DH and DPPH, negative signs for A, C and D coefficients gave negative effects, while a positive sign for B coefficient gave a positive effect towards FRAP activity (Equation (8)). This implies that the FRAP response was inversely proportional to the pH (A), reaction time (C) and E/S ratio (D), while directly proportional to the reaction temperature (B).

**Figure 1:**
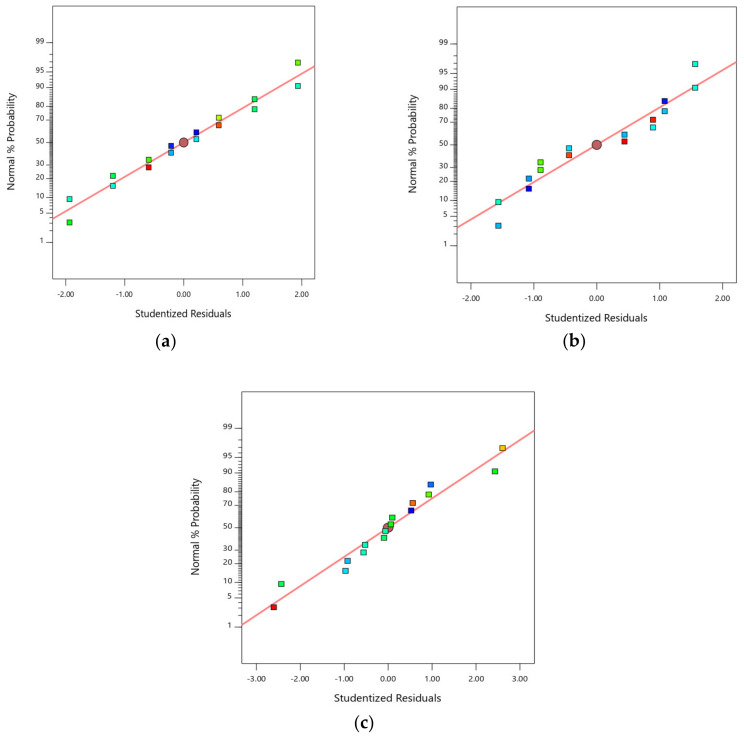
Normal plot of residual for (**a**) degree of hydrolysis (DH), (**b**) 2,2-diphenyl-1-picrylhydrazyl (DPPH) radical scavenging activity and (**c**) ferric-reducing antioxidant power (FRAP) activity.

Figure 1 provides the normal plot of the residuals of the three responses. The plot was used to verify the normality assumption of the data. Residuals are a different value measured between the experimental and theoretical. If the data plot falls closely to the straight line, the data is normally distributed [6,18]. As shown from the graph, all responses had data points that were fairly close to the straight line, with no outlier points. These revealed that the experiments come from a normally distributed population.

### 3.2. Effect of Parameters on Degree of Hydrolysis

**Figure 2:**
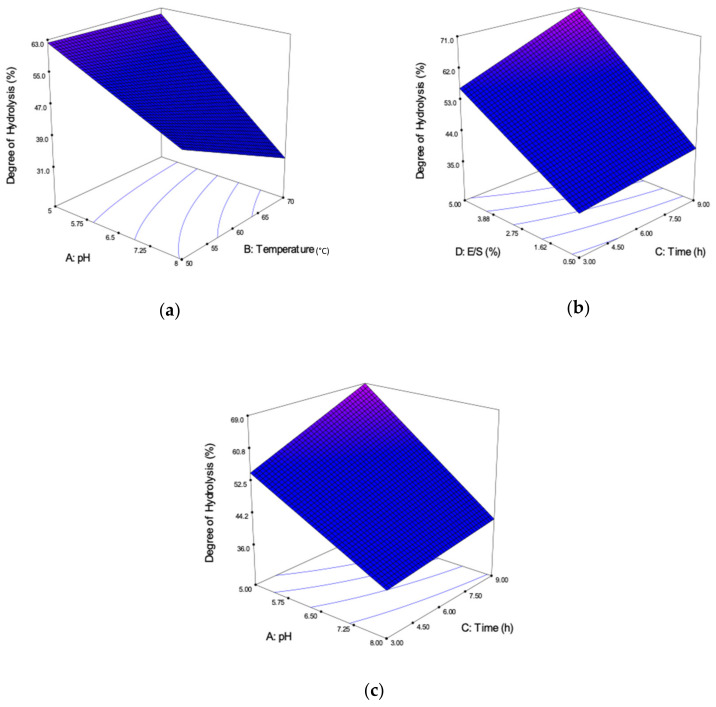
Three-dimensional (3D) contour plots showing the influence of variable parameters on the degree of hydrolysis (DH) response. (**a**) pH vs. reaction temperature, (**b**) reaction time vs. E/S ratio and (**c**) pH vs. reaction time.

Generally, the hydrolysis of protein is measured in terms of DH, which is defined as the percent ratio of the number of peptides dissociated to the total number of peptide bonds in the substrate. Figure 2 shows the three-dimensional (3D) model graph interaction effects on the DH response. These graphs were generated from the significant interaction effects in the ANOVA.

Figure 2a correlates the interaction effect between the pH (A) and reaction temperature (B) at a constant time of 6 h and 2.75% E/S ratio (D). Based on the model graph, the highest DH was found at a lower pH (pH 5.00) and temperature (50 °C). At pH 5.00, increasing the temperature does not affect the DH significantly. Theoretically, the DH is affected by the activity of the papain enzyme, depending on the suitability of the pH medium [26,28]. As a result, increasing the pH from acidic (pH 5.00) to base (pH 8.00) will decrease the DH. Hence, the suitable pH at this reaction condition was pH 5.00.

Figure 2b represents the combined effects between reaction time (C) and E/S ratio (D) at a constant pH 6.50 and 50 °C temperature. The highest DH was obtained at a 5.00% E/S ratio and longer reaction time of 9 h. High E/S ratio will raise the amount of amino acid peptides cleaved by the papain enzyme; more enzymes, more cleaved peptides, thus increasing the DH [16]. Moreover, an increase in time will increase the DH, as there was enough time for the proteins to be cleaved [26].

Last of all, Figure 2c shows the interaction between pH (A) and reaction time (C) at a fixed temperature of 60 °C and a 2.75% E/S ratio. Comparable to the interaction of AB, the highest DH also can be achieved at pH 5.00 but with a longer reaction time of 9 h. Therefore, decreasing the time will decrease the amount of amino acid cleaved. Hence, 9 h was the suitable reaction time needed in this reaction.

**Figure 3:**
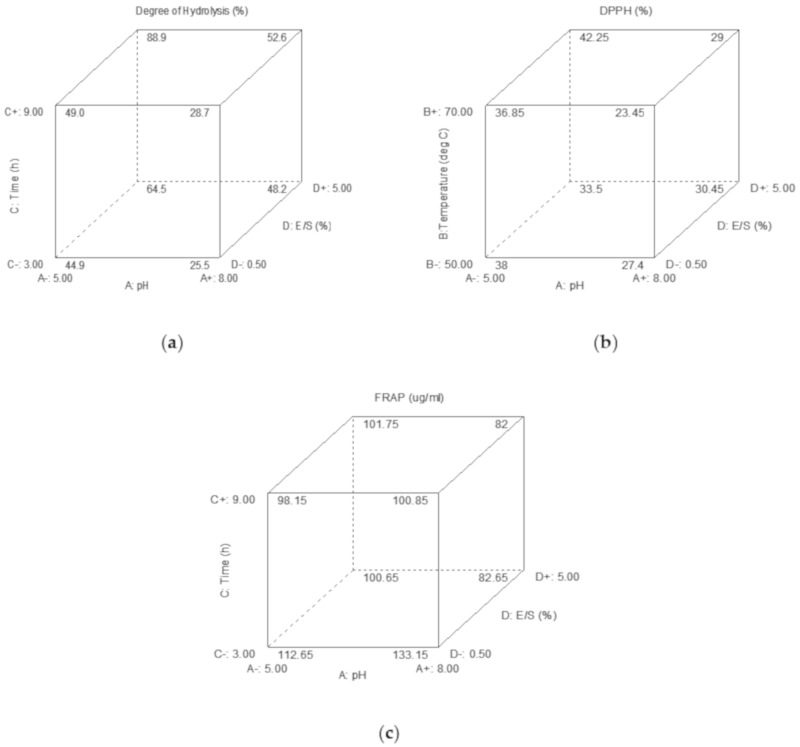
Cube plots showing the interaction of three significant effects on (**a**) the degree of hydrolysis (DH) response, (**b**) 2,2-diphenyl-1-picrylhydrazyl (DPPH) radical scavenging response and (**c**) ferric-reducing antioxidant power (FRAP) response.

Figure 3a represents a cube plot showing the combination of three significant factors on the DH response. The interaction factors are ACD (pH, reaction time and E/S ratio). At each corner, the highest and lowest value of each effect is presented. The negative sign indicates the lower limit, while the positive sign indicates the higher limit [29]. According to the represented graph, DH is maximum at A − (pH 5.00), C + (9 h) and D + (5.00 % E/S). This result was in agreement with the ANOVA in Table 3, in which the ACD was the combination of significant factors, excluding the reaction temperature (B).

### 3.3. Effect of Parameters on Antioxidant Activities

#### 3.3.1. DPPH Radical Scavenging Activity

**Figure 4:**
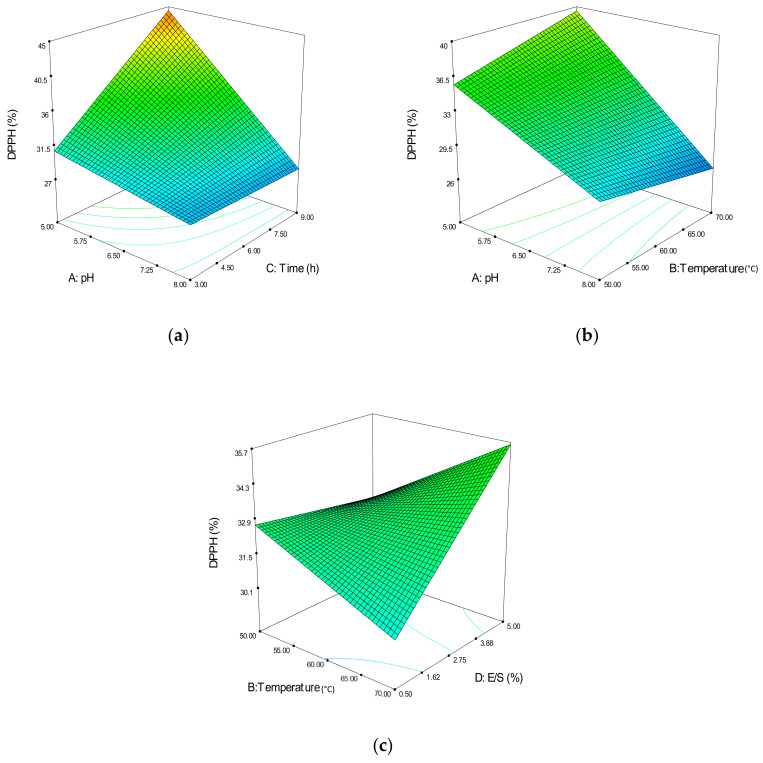
3D contour plots showing the influence of variable parameters on the 2,2-diphenyl-1-picrylhydrazyl (DPPH) radical scavenging response. (**a**) pH vs. reaction time, (**b**) pH vs. reaction temperature and (**c**) reaction temperature vs. E/S ratio.

Evaluation of the antioxidant activity for compounds that act as free radical scavengers or hydrogen donors can be tested using a relatively stable DPPH radical. The DPPH radical scavenging activity of hydrolysates from *A. lecanora* are presented in Figure 4.

As shown in Figure 4a, the interaction between pH (A) and reaction time (C) was plotted at a fixed 60 °C temperature and 2.75% E/S ratio. Higher DPPH radical scavenging activity can be achieved at pH 5.00 with a maximum reaction time of 9 h. This result was in conjunction with Ghanbari et al. [14], who stated that a higher DPPH scavenging activity could be obtained with a longer reaction time, approximately more than 8 h.

Figure 4b indicates the interaction effects between pH (A) and reaction temperature (B). A higher DPPH radical scavenging activity was also achieved at pH 5.00, with a higher temperature, 70 °C, which indicates the suitable condition for the papain enzyme in this study. This is in agreement with the optimal pH condition for the papain enzyme suggested by Kusumadjaja et al. [30], with pH 5.00–7.00, and Singh et al. [31], which appears to be in the range of pH 4.00–6.00. The optimal conditions for the temperature are slightly higher (70 °C) compared to the described condition of 50–65 °C [32], mostly because of the different hydrolysis preparation. However, in another work, Noman et al. [33] reported that the optimum temperature for the protein hydrolysates prepared from the muscles of a Chinese sturgeon was at 70 °C.

Another significant interaction effect designated in Figure 4c indicates the interaction effects between the reaction temperature (B) and E/S ratio (D). At 70 °C, increasing E/S ratio will increase DPPH radical scavenging activity. Prior studies have reported that the antioxidant activities can be increased through certain enzymes with certain E/S ratios [28]. Thus, increasing the E/S ratio might offer more suitable hydrolysates for the DPPH radical scavenging activity.

The cube plot in Figure 3b summarizes the interaction of three significant factors of ABD, which were the pH, reaction temperature and reaction time. The highest DPPH responses were at A − (pH 5.00), B + (70 °C) and D+ (5.00% E/S ratio). This outcome was also parallel with the ANOVA and 3D response graph mention above.

#### 3.3.2. Ferric-Reducing Antioxidant Power Activity

**Figure 5:**
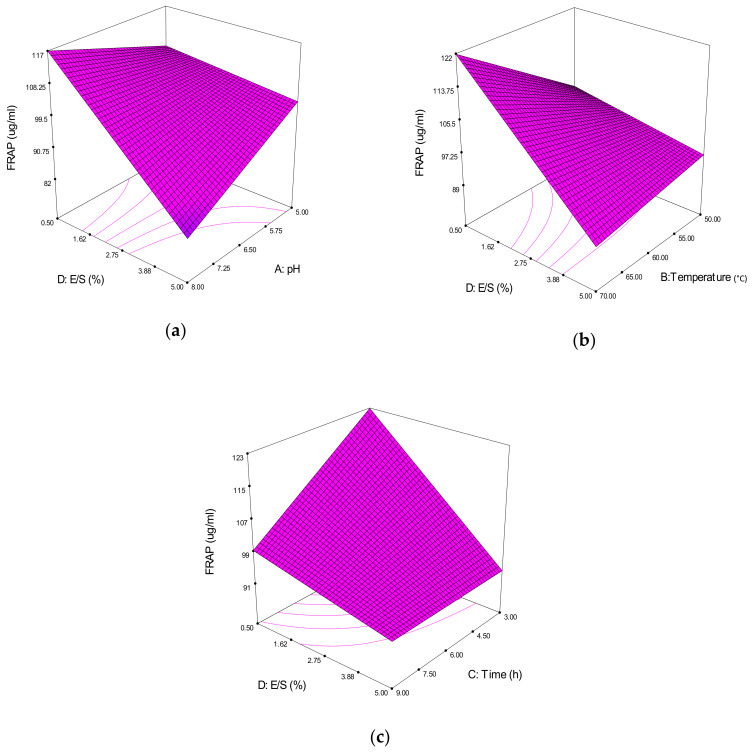
3D contour plots showing the influence of variable parameters on the ferric-reducing antioxidant power (FRAP) response. (**a**) pH vs. E/S ratio, (**b**) reaction temperature vs. E/S ratio and (**c**) reaction time vs. E/S ratio.

The FRAP method is used to measure the capacity of the substance in reducing the TPTZ-Fe^3+^ yellow color complex to the TPTZ-Fe^2+^ blue color complex. This action was performed by the action of electron-donating antioxidants [34]. From the 3D model graph, Figure 5a shows the interaction effects between the pH (A) and E/S ratio (D). Higher FRAP can be achieved at the 0.50% E/S ratio with a higher pH 8.00. At pH 8.00, the FRAP value was inversely proportional to the E/S ratio. This showed that adding more enzymes at an inappropriate pH does not increase the FRAP activity.

Figure 5b shows the interaction effect between the reaction temperature (B) and E/S ratio (D). From the 3D plot, a higher FRAP is also at 0.50% E/S, with a higher temperature (70 °C). This result was in conjunction with the DPPH radical scavenging activity, which had a similar reaction temperature to achieve its highest yield.

The interaction effects between the reaction time (C) and E/S ratio (D) were revealed in Figure 5c. Higher FRAP is also at the 0.50% E/S ratio, with only a minimum reaction time of 3 h. A study of mungbean meal protein hydrolysates found out that the FRAP activity of peptide fractions increased with increases in the molecular weight of the peptides [35]. This implied that the short reaction time might influence the molecular weight of the peptides, and a lower reaction time will produce a larger chain with a higher molecular weight. However, the study also stated that the molecular weight might not be the most important contributing factor, since the amino acid in the peptide chain and sequence also had influence on the FRAP activity.

Overall, a higher FRAP activity was mostly obtained at a lower E/S ratio of 0.50% in conjunction with a higher reaction temperature of 70 °C. The higher FRAP activity suggested that the hydrolysates have a high reducing power that could donate an electron to free radicals, hence leading to the prevention or retardation of propagation [36].

As for the cube plot in Figure 3c, the combination factors were also from ACD, with the highest predicted FRAP values pointed at A + (pH 8), C − (3 h) and D − (0.50% E/S ratio). FRAP reaction conditions might be differ from the DPPH response mainly because of their different mechanisms. FRAP activity favors the higher molecular weight of peptides, while DPPH activity fancies the lower molecular weight of peptides.

### 3.4. Validation of the Model and Optimum Condition for Hydrolysis

The actual and predicted responses for model validations are represented in Table 5. Several sets of experiments were conducted by randomly changing the variables. These model validation sets were performed to determine the adequacy of the final model. Results show insignificant differences between actual and predicted values, by comparing the residual standard error (RSE) percentage value lower than ± 5.00% (Equation (2)). This proved that the models suggested were adequate.

The optimum combinations of parameters were generated by fixing all parameters, including the DH responses in the range, while maximizing the DPPH radical scavenging activity and FRAP activity responses. By considering the need to lower the production costs, a parameter such as the E/S ratio was minimized, while the pH, reaction temperature and time were set to within the range. Based on those settings, one optimized condition of maximum desirability (D close/equal to 1) was selected. Sample O1 was hydrolyzed at pH 5.00, 70 °C reaction temperature, 9 h hydrolysis time and with a 1.00% E/S ratio. The optimized sample was in good agreement with the predicted values, with an RSE value of 1.60%.

### 3.5. Amino Acid Profiles of the Optimized A. lecanora Hydrolysates

The amino acids composition of the optimized *A. lecanora* hydrolysates are shown in Table 6. The total amount of hydrophobic amino acid was higher than the total amount of hydrophilic amino acids, with 286.40 mg/g sample and 253.60 mg/g sample, respectively. As for the hydrophobic amino acid composition, glycine was the most abundant (86.20 mg/g sample), followed by proline, alanine, leucine, valine, isoleucine, tyrosine, phenylalanine and methionine. In the hydrophilic amino acid composition, glutamic acid dominated among others with 84.40 mg/g sample, which was three-fold the amount of aspartic acid. These were followed by arginine, threonine, serine and histidine.

The presence of hydrophobic amino acids was responsible for the antioxidant activity of the hydrolysates [14]. For example, in the DPPH radical scavenging activity, aromatic amino acids such as tyrosine, histidine, tryptophan and phenylalanine, together with hydrophobic amino acids such as valine, leucine, alanine and methathione, play the most important role. The presence of these amino acids in the peptide sequence will increase its access to reactive free radicals and increase the solubility of the peptides in lipids, hence enhancing their ability to generate antioxidative activity [14,24,37]. Moreover, these amino acids result in a higher efficacy when located at the C-terminal compared to the N-terminal region [26,38].

In a previous study, Zhuang et al. [37] found that the amino acid compositions of the three fractions from jellyfish (*Rhopilema esculentum*) hydrolysates were rich in glycine, glutamic acid, proline, aspartic acid, valine and arginine related to its antioxidant activity. Guo et al. [39] discovered that most of the peptides sequences identified in armored catfish (*Pterygoplichthys disjunctivus*) hydrolysates contain several acidic amino acids in their sequences; glutamic acid and aliphatic amino acids (alanine, isoleucine and leucine) were related to the highest FRAP activity.

Therefore, the presence of appreciable amounts of hydrophobic amino acids and hydrophilic amino acids in *A. lecanora* hydrolysates can be related to its antioxidant activity. Furthermore, the composition of amino acids for papain-generated *A. lecanora* hydrolysates was in good agreement to the study conducted by Ghanbari et al. [14].

### 3.6. Scanning Electron Microscpy

**Figure 6:**
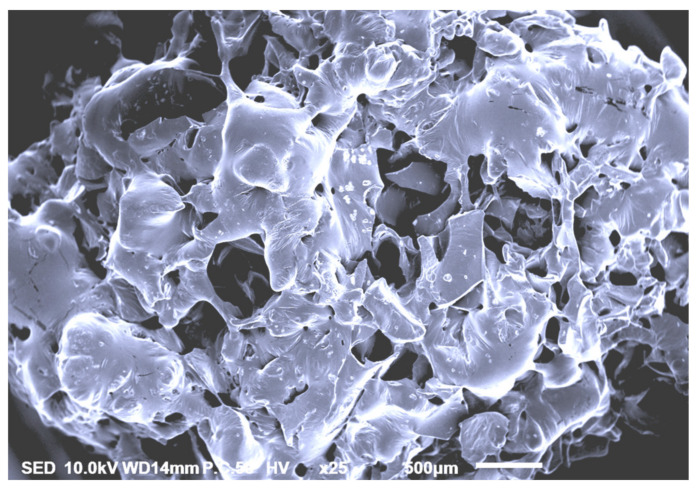
Scanning electron microscopy (SEM) image of papain-generated hydrolysates from *Actinopyga lecanora*.

The SEM image for optimized *A. lecanora* hydrolysates showed a smooth structure with pores (Figure 6). A similar smooth vesicular pattern was reported from the peptide sequence of Glu-Pro-Ala-His, generated from *Auxis thazard* hydrolysates [40]. Meanwhile, the porous form indicates that the protein was degraded into smaller peptides. Previous works performed with marine sources revealed that the molecular weights of peptides influence the morphology of the hydrolysates [41]. The smaller the peptides, more pores will be formed and increase the open structure in the image. León-López et al. [42] found out that SEM images of the hydrolyzed collagen from sheepskin had a porous and spongy structure, which the degradation of the protein can be seen upon increasing the hydrolysis time.

### 3.7. Tyrosinase Inhibition Activity

Tyrosinase inhibition activity was tested for the optimized *A. lecanora* hydrolysates sample using L-DOPA and *L*-tyrosine as substrates. Both substrates were associated to the reaction mechanism of monophenolase activity and diphenolase activity. In monophenolase activity, the monophenols (e.g., L-tyrosine) hydroxylates to *o*-diphenols (e.g., L-dopa), while the diphenolase activity involving tyrosinase oxidizes *o*-diphenols to *o*-quinones (*o*-dopaquinone) [43].

The ability to inhibit tyrosinase activity might be related to the presence of hydrophobic and aliphatic amino acids, like valine, alanine and leucine. In addition, amino acids with a hydroxyl group such as serine and threonine are also related to the antityrosinase activity [6]. Prakot et al. [44] reported that the spotted Babylon powder hydrolysates contained small amounts of tyrosine, with 16.60 mg/g, quite similar to the amount of tyrosine from the *A. lecanora* hydrolysates (17.00 mg/g). Low amounts of tyrosine are favorable, because it is one of the substrates in melanin synthesis. Therefore, large amounts of tyrosine in the protein hydrolysates might increase substrate concentrations and reduce the inhibitory activity.

**Figure 7:**
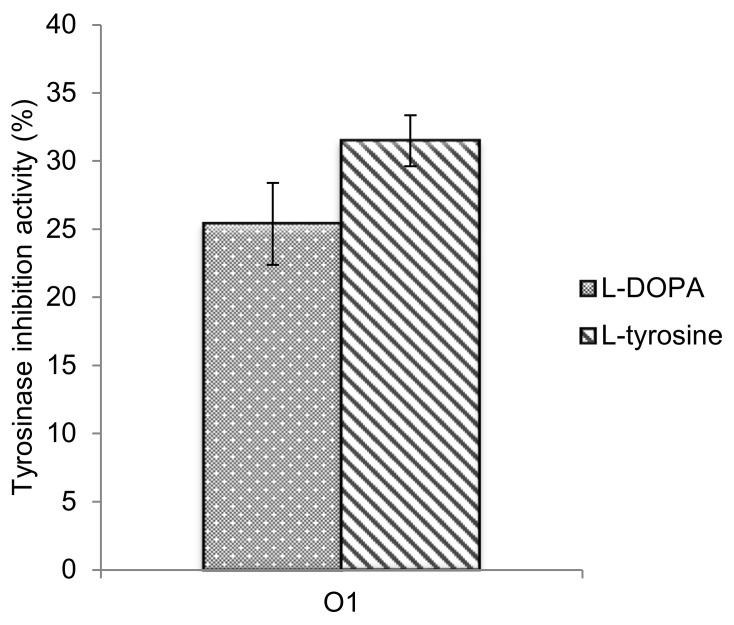
Tyrosinase inhibition activity for an optimized *A. lecanora* hydrolysates sample.

Based on Figure 7, 31.50% and 25.40% of tyrosinase inhibition activity were recorded when using L-tyrosine and L-DOPA substrates, respectively. The activity was tested at 10 mg/mL. Wu et al. [45] recorded that the inhibitory effects on tyrosinase of sericin hydrolysates was more than 50.00% with the L-DOPA substrate. Zhuang et al. [37] reported that the tyrosinase inhibitory activity fractions from jellyfish (*Rhopilema esculentum*) were 21.70%–53.90% at 5mg/mL also when using the L-DOPA. Although our results exhibited lower tyrosinase inhibitory activity, it is still potent in expanding other functions of the *A. lecanora* hydrolysates, as, so far, no related tyrosinase inhibition activity has been documented for this species. Besides, different tyrosinase inhibition methods might also influence the findings; for example, crude hydrolysates revealed a lower tyrosinase inhibition activity compared to the peptide fractions [46].

After all, other researchers have mentioned that proteins and peptides from natural resources such as honey, wheat, milk and silk are capable of inhibiting tyrosinase activity [5,22,37]. Therefore, the papain-generated hydrolysates produced from this study could be one of the natural tyrosinase inhibitors that can be utilized in cosmeceutical industries.

## 4. Conclusions

The present study demonstrated that the optimization of the papain enzymatic hydrolysis of *A. lecanora* was successfully performed using two-levels of factorial design. The selected optimized conditions were at pH 5.00, 70 °C reaction temperature, 9 h hydrolysis time and with a 1.00% E/S ratio that gave 51.90% DH, 42.70% DPPH scavenging activity and 109.9 Fe^2+^μg/mL reducing power in a FRAP assay. All models for Y_1_, Y_2_ and Y_3_ responses were significant, with R^2^ of 99.48%, 99.79% and 99.68%, respectively. The amino acid composition of the optimized papain-generated hydrolysates showed that the presence of hydrophobic amino acids were responsible for the antioxidant and antityrosinase activity. The SEM image displayed the morphology of the optimized hydrolysates as smooth structures with pores. The tyrosinase inhibitions for the optimized sample gave 31.50% and 25.40% at 10 mg/mL in the L-tyrosine and L-DOPA substrates, respectively. As a whole, these results suggested that the optimized papain-generated *A. lecanora* hydrolysates can be considered as one of the natural sources of antioxidant and antityrosinase products in cosmeceutical industries.

## Figures and Tables

**Table 1:** Experimental ranges and independent variables used in enzymatic hydrolysis optimization.

Independent Variable	Coded Variable	Variable Levels	
		Low (−1)	High (+1)
pH	A	5.00	8.00
Reaction temperature, °C	B	50	70
Reaction time, t	C	3	9
Enzyme/substrate ratio (E/S), %	D	0.50	5.00

**Table 2:** Design matrix and responses of a two-level factorial design.

Run	Variables				Responses					
	A	B	C	D	DH (%)		DPPH (%)		FRAP (Fe^2+^μg/mL)	
					Actual	Predicted	Actual	Predicted	Actual	Predicted
1	5	50	3	0.50	40.40	42.00	28.50	28.70	92.50	93.10
2	5	50	3	5.00	32.00	32.40	26.90	27.30	126.40	124.70
3	5	70	3	0.50	49.30	47.70	27.70	27.50	132.80	124.70
4	5	70	3	5.00	19.10	18.80	23.70	23.30	132.80	132.10
5	8	50	3	5.00	47.80	46.20	47.50	47.30	139.90	141.60
6	8	50	3	0.50	35.90	35.60	27.90	27.50	84.30	85.30
7	8	70	3	0.50	50.20	51.80	46.00	46.20	112.00	111.00
8	8	70	3	5.00	21.60	21.90	23.20	23.60	102.80	102.70
9	5	50	9	5.00	69.20	68.30	29.50	29.20	94.30	94.40
10	5	50	9	0.50	54.60	56.70	30.80	31.30	86.40	87.40
11	5	70	9	0.50	59.90	60.80	37.60	37.90	107.00	106.90
12	5	70	9	5.00	41.90	39.80	30.30	29.80	78.90	77.90
13	8	50	9	0.50	91.70	92.60	37.50	37.80	103.80	102.10
14	8	50	9	5.00	63.20	61.10	30.10	29.60	91.80	93.40
15	8	70	9	0.50	86.10	85.20	46.90	46.60	99.70	101.40
16	8	70	9	5.00	42.00	44.10	27.70	28.20	71.20	70.60

A: pH. B: reaction temperature (°C). C: reaction time (t). D: enzyme/substrate (E/S) ratio (%). DH: degree of hydrolysis. DPPH: 2,2-diphenyl-1-picrylhydrazyl. FRAP: ferric-reducing antioxidant power.

**Table 3:** Results of ANOVA for different responses. **R^2^**: coefficient of determination.

Responses	Coefficients	*p*-Value	Std. Dev.	% C.V.	Mean Value	R^2^	Pred R^2^	F Value	Significance
**DH (Y_1_)**									
A	−11.52	<0.0001							
B	−4.04	0.0048							
C	4.51	0.0032							
D	13.27	<0.0001	2.85	5.67	50.31	0.9948	0.9162	69.08	0.0005
AB	−3.59	0.0073							
AC	−2.62	0.0213							
BD	−2.06	0.0448							
CD	2.67	0.0201							
ACD	−2.38	0.0228							
**DPPH (Y_2_)**									
A	−5.04	<0.0001							
C	3.24	<0.0001							
D	1.19	0.0026							
AB	−1.63	0.0008							
AC	−3.59	<0.0001	0.71	2.17	32.61	0.9979	0.9667	174.48	< 0.0001
AD	0.96	0.0056							
BD	1.55	0.0009							
CD	−1.49	0.0011							
ABD	−0.93	0.0064							
ACD	1.01	0.0046							
**FRAP (Y_3_)**									
A	−1.82	0.0246							
B	4.06	0.0014							
C	−5.79	0.0004							
D	−9.72	<0.0001							
AB	−5.52	0.0004							
AC	−2.44	0.0092	2.07	2.04	101.48	0.9968	0.9491	113.87	0.0002
AD	−7.62	0.0001							
BC	−3.32	0.0030							
BD	−6.62	0.0002							
CD	5.91	0.0003							
ACD	2.01	0.0179							

**Table 4:** Quadratic polynomial equations for the three responses.

Responses	Equations	
DH	Y_1_ = 50.31 − 11.52A − 4.04B + 4.51C + 13.27D − 3.59AB − 2.62AC − 1.63AD − 2.06BD + 2.67CD − 2.38ACD	Equation (6)
DPPH radical scavenging activity	Y_2_ = 32.61 − 5.04A + 3.24C + 1.19D − 1.63AB − 3.59AC + 0.96AD + 1.55BD − 1.149CD − 9.93ABD + 1.01ACD	Equation (7)
FRAP activity	Y_3_ = 101.48 − 1.82A + 4.06B − 5.79C − 9.72D − 5.52AB − 2.44AC − 7.62AD − 3.32BC − 6.62BD + 5.91CD + 2.01ACD	Equation (8)

**Table 5:** Actual and predicted responses for the model verification.

Set	Variables				DH (%)			DPPH (%)			FRAP (Fe^2+^μg/mL)		
	A	B	C	D	Actual	Predicted	RSE	Actual	Predicted	RSE	Actual	Predicted	RSE
V1	5.00	70	7	0.50	50.00	50.40	0.80	39.30	40.00	1.70	112.50	118.10	4.70
V2	8.00	70	8	1.00	53.90	53.40	−0.90	39.40	40.40	2.50	115.10	116.40	1.10
V3	8.00	70	7	1.00	52.40	52.30	−0.20	36.20	37.50	3.30	118.00	119.60	1.30
V4	8.00	70	6	1.00	54.50	54.50	0.00	41.70	43.30	3.70	113.20	113.20	0.00
O1	8.00	70	9	1.00	55.00	55.50	0.90	46.30	46.20	−0.20	108.20	110.00	1.60

**Table 6:** Amino acid composition (mg/g dry weight) of optimized papain-generated *Actinopyga lecanora* hydrolysates.

Type of Amino Acid	Amino Acid	Papain-Generated Hydrolysates (mg/g Sample)
Hydrophilic	Glutamic acid (E)	84.40
	Aspartic acid (D)	51.10
	Arginine (R)	45.30
	Threonine (T)	29.20
	Serine (S)	22.10
	Lysine (K)	17.20
	Histidine (H)	4.30
Total		253.60
Hydrophobic	Glycine (G)	86.20
	Proline (P)	44.10
	Alanine (A)	43.90
	Leucine (L)	30.60
	Valine (V)	25.60
	Isoleucine (I)	18.90
	Tyrosine (Y)	17.00
	Phenylalanine (F)	15.60
	Methionine (M)	4.90
Total		286.40
Total hydrophilic and hydrophobic		540.00
